# Open abdomen management for massive intestinal infarction due to acute splanchnic venous thrombosis in a patient with protein S deficiency. A case report

**DOI:** 10.1016/j.ijscr.2020.05.076

**Published:** 2020-06-06

**Authors:** Annamaria Di Bella, Alessandro Bruscino, Giovanni Alemanno, Carlo Bergamini, Paolo Prosperi

**Affiliations:** Emergency Surgery Unit, Careggi University Hospital, Largo Brambilla 3, 50134, Florence, Italy

**Keywords:** AMI, Acute Mesenteric Ischemia, PMVS, Porto-Mesenteric Venous System, OA, Open abdomen, CECT, Contrast-Enhanced Computed Tomography, HP, Helycobacter Pylori, ICU, Intensive Care Unit, Open abdomen, Splanchnic thrombosis, Portomesenteric, Thrombosis, Protein s deficiency, Intestinal infarction, Thrombophilia

## Abstract

**Introduction:**

Acute mesenteric ischemia (AMI) refers to the sudden onset of intestinal hypoperfusion that can also result from splanchnic venous occlusion. The portomesenteric venous system (PMVS) is an unusual site of thrombosis in patients with protein S deficiency and its obstruction is a rare cause of AMI. Aim of this report is to illustrate a successful strategy in a case of massive small bowel infarction managed with an open abdomen (OA) approach.

**Case presentation:**

A 64 year-old woman presented to the emergency department with acute abdominal pain, rectal bleeding, diarrhea and vomiting. Contrast-enhanced computed tomography (CECT) showed small bowel ischemia and the complete occlusion of all the PMVS branches. Surgery was performed with an OA approach and anticoagulation was immediately begun. Further workup revealed isolated protein S deficiency and history of atrophic gastritis. Thromboprophylaxis with warfarin was started on discharge and no recurrence of thrombotic events was recorded during the one-year follow-up.

**Discussion:**

PMVS thrombosis related to protein S deficiency is a rare condition that can rapidly lead to an acute abdomen. CECT is the gold standard, because it detects splanchnic thrombosis and its possible complications, like bowel ischemia. In case of surgery, a planned second-look operation is the best strategy to assess bowel viability and possible ischemic progression.

**Conclusions:**

OA management plays a fundamental role in case of resection for bowel ischemia. Patients with thrombosis at an uncommon site should be further investigated for prothrombotic states.

## Highlights

• Portomesenteric venous thrombosis is rare and can lead to serious consequences.
• Open abdomen is a successful strategy in case of resections for bowel ischemia.
• Thrombosis at an ucommon site should be investigated for prothrombotic states.
• Long-term anticoagulation is recommended in lifelong trombophilic states.

## 1 Introduction

Porto-Mesenteric Venous System (PMVS) thrombosis is an uncommon but potentially lethal condition that is extremely rare among non oncologic and non cirrhotic patients [1]. The reported incidence is 2,7 per 100,000 person-years [2]. Despite recent diagnostic and therapeutic advances, the still poor prognosis associated with PMVS thrombosis is due to its possible progression to intestinal infarction. The hospital mortality is between 59 and 93 % [3].

The clinical presentation is variable, ranging from asymptomatic to non specific abdominal pain to acute abdomen.

The gold standard for its detection is Contrast-Enhanced Computed Tomography (CECT), which also can reveal other associated conditions like bowel ischemia [4].

PMVS thrombosis is generally caused by the association of inherited or acquired prothrombotic conditions and local causes, but sometimes its origin remains idiopathic.

Protein S is a vitamin K dependent anticoagulant. Its deficiency can present with recurrent episodes of venous thromboembolism and its prevalence in patients with splanchnic vein thrombosis is 2,6 % [5]. Trombophilia due to protein S deficiency can be inherited with an autosomal dominant pattern, and the entity of the deficit can be mild or severe depending on whether the mutation is heterozygous or homozygous.

In this paper, is presented a case of massive small bowel infarction due to acute PMVS thrombosis in a 64 year-old woman with history of chronic athrophic gastritis and protein S deficiency, successfully treated with a multidisciplinary approach based on the surgical open abdomen management.

This study has been reported in line with the SCARE criteria [6].

## 2 Case presentation

A 64 year-old woman presented to the emergency department complaining of severe diffuse abdominal pain, associated with multiple episodes of vomiting, rectal bleeding and diarrhea. Pain was acute, dull, non-radiating, increasing in severity in the last 24 h. She had no relevant medical history except for a microcytic anemia and an atrophic gastritis with chronic Helycobacter Pylori (HP) infection. The physical examination revealed abdominal distention and rebound tenderness in epigastrium and right quadrants, positive Blumberg’s sign and absence of bowel sounds.

Blood tests showed a white blood cell count of 13,600/mm^3^, an hemoglobin of 8,5 g/dL with a mean corpuscolar volume of 582 fL, prothrombin time/international normalized ratio of 17,8 s/1,3, protein level of 3,5 g/dL and albumin of 21 g/L.

Arterial blood gas analysis showed 2,7 mMol/L (ref. 0,9−1,7 mMol/L) lactate acid level.

**Fig. 1:**
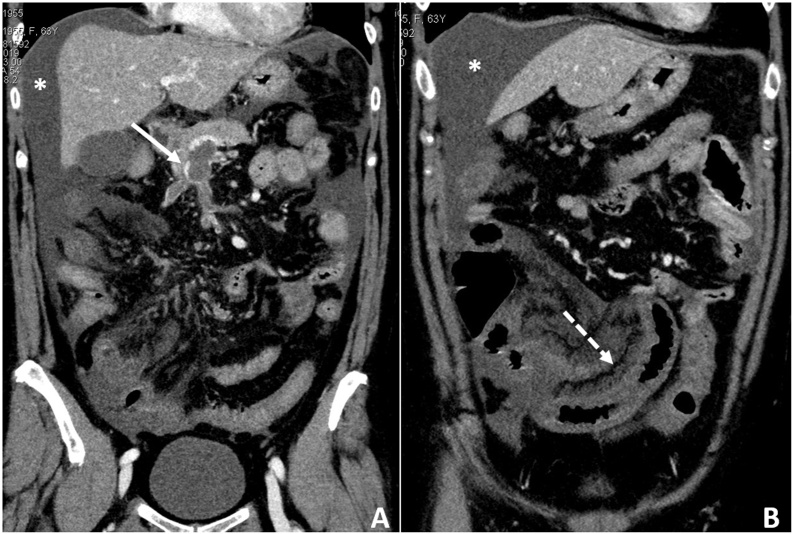
**A, B** Coronal reconstruction of CECT scan showing enlargement of portal vein and superior and inferior mesenteric veins (A, arrow) with no contrast enhancement due to acute thrombosis. Thickening and absence of enhancement of bowel walls due to venous ischemia (dotted arrow), with mesenteric edema. Presence of abundant free peritoneal fluid, mostly perihepatic (A–B, asterisk).

An US examination showed free abdominal fluid and portal vein thrombosis. Hence a CECT was performed and revealed a massive thrombosis of right intrahepatic portal branches, portal vein, superior and inferior mesenteric veins with associated extended acute venous ischemia of the small bowel, colonic wall thickening with normal contrast enhancement and abundant peritoneal effusion (Fig. 1).

The patient was referred to the emergency surgery department. An explorative laparotomy revealed the presence of abundant bloody ascitic fluid and edema of the mesentery and of the entire small bowel; furthermore evidence of venous infarction of about 130 cm of the terminal ileum was found, and thus surgical resection was performed. The bowel was left in discontinuity and an open abdomen management was adopted to better assess intestinal viability. After surgery, the patient was transferred to the Intensive Care Unit (ICU). Antibiotic prophylaxis, blood transfusions, total parenteral support nutrition, as well as anticoagulation with low-molecular-weight heparin infusion were immediately initiated. The second look surgery was planned 48 h after the first operation and a terminal ileostomy was performed. The patient was extubated the following day and discharged from ICU two days after extubation.

The following work-up showed no evidence of malignancies or other acquired risk factors for PMVS thrombosis except for an atrophic gastritis with chronic HP infection. Investigations to detect any prothrombotic disease revealed only a mild protein S deficiency.

The patient was discharged from the hospital on the 15th postoperative day, after switching anticoagulant therapy on warfarin; new oral anticoagulants could not be administered for an acute kidney injury likely due to dehydration because of the ileostomy. Besides, the patient had begun the HP eradication therapy.

**Fig. 2:**
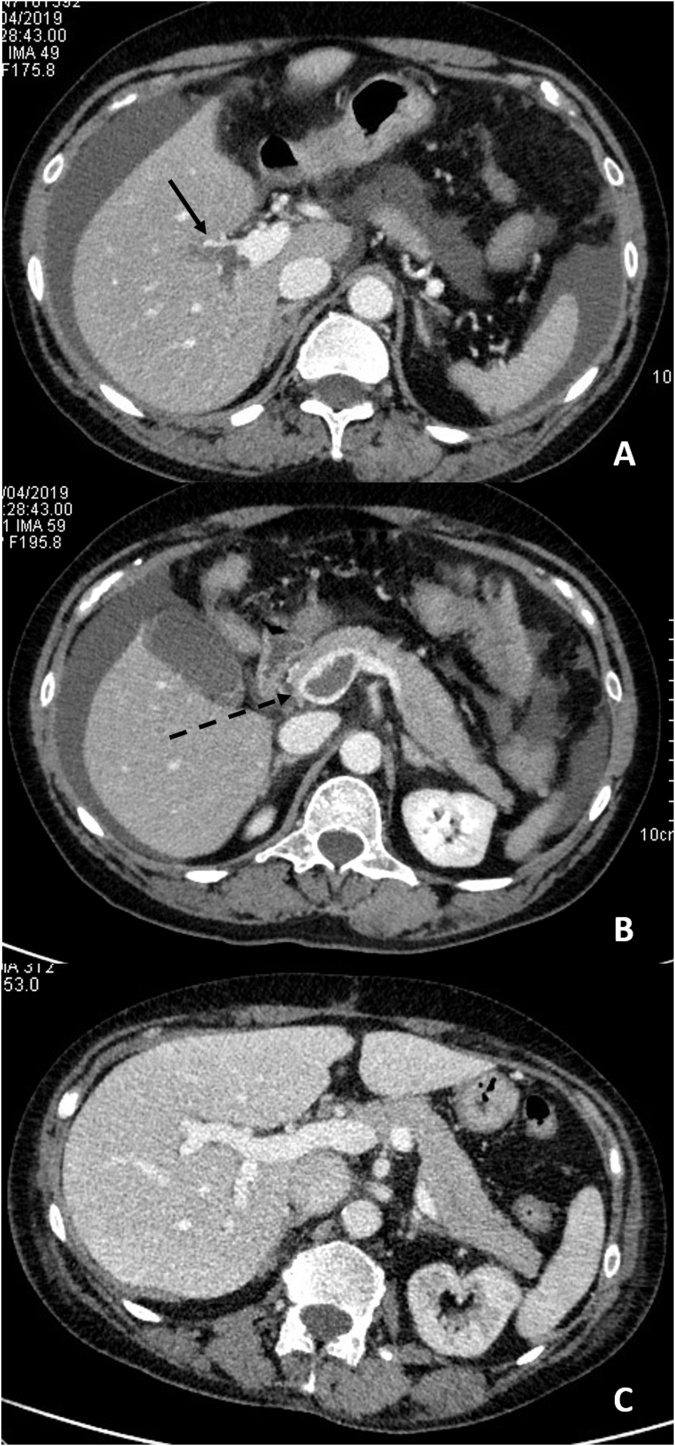
**A, B, C** Comparison between initial (A, B) and follow-up (C) CECT examinations. Axial views showing acute thrombosis of the main intrahepatic branches of portal vein (A, arrow) and of the extrahepatic portal vein (B, dotted arrow). Follow-up axial image demonstrating complete resolution of the thrombosis (C, arrowhead) and of the ascites.

After three months, the PMVS thrombosis was completely resolved (Fig. 2) and the patient could undergo surgery to have her bowel continuity restored.

At the 1-year follow-up, the patient was in good clinical condition, with no further episodes of thrombosis, HP eradication confirmed at gastric biopsy and improvement of microcytic anemia and creatinine levels.

## 3 Discussion

The PMVS refers to the vessels involved in the drainage of the gastrointestinal tract and spleen and it’s composed by superior and inferior mesenteric veins, portal vein and splenic vein.

PMVS thrombosis is a rare disease, with a global mortality rate of 20–50 % [2]. The diagnosis may be difficult due to the variable clinical presentation depending on thrombosis extension and onset timing.

CECT is the technique of choice in detecting both filling defects inside the venous vessels and ischemic changes in the bowel [4].

PMVS thrombosis is often the result of a combination of local and systemic risk factors that could be divided into inherited or acquired (Table 1).

**Table 1:** PMVS THROMBOSIS RISK FACTORS.

INHERITED RISK FACTORS	
COMMON	UNCOMMON
Factor V Leyden mutation	Antithrombin III deficiency
Factor II mutation [G20210A]	Protein C deficiency
MTHF reductase gene mutation - [C677 T] - [A1298C]	Protein S deficiency

ACQUIRED RISK FACTORS	
COMMON	UNCOMMON
Cirrhosis and Hepatocarcinoma	Myeloproliferative diseases [JAK2, CALR, MPL mutations]
Oral contraceptive assumption	Antiphospholipid syndrome
Pregnancy and post-partum	Paroxysmal nocturnal hemoglobinuria
Obesity and smoke	
GI Inflammatory conditions (chronic and acute): - pancreatitis - cholecystitis - diverticulitis - IBD	
Protein-losing conditions: - nephritic syndrome - protein-losing gastro-enteropathy	
Malignancies - paraneoplastic syndrome - direct infiltration - abdominal mass compression Chemotherapy	
Hyperhomocysteinemia	
Surgery - laparoscopy - obesity surgery - transplants - gastrectomy, cholecystectomy, splenectomy, etc.	

Most common inherited risk factors for PMVS thrombosis have a prevalence >2% in general population and carry a relatively low risk of splanchnic thrombosis, while more rare conditions, like protein S deficiency, with a prevalence <0,4 %, are associated with a higher relative risk of thrombosis [7].

Protein S is a plasma serine protease involved in coagulation, inflammation and apoptosis and its deficiency results in thrombophilia. This condition is usually congenital, caused by more than 200 different mutations in the PROS1 gene [8], leading to various degrees of protein S levels and/or activity. However, there are many acquired conditions that can cause protein S level fluctuations, such as Vitamin K antagonist therapy, chronic infections, severe hepatic diseases, nephritic syndromes and Disseminated Intravascular Coagulation [9].

In the presented case, the patient's medical history and the diagnostic tests allowed to excludemalignancies or other acquired risk factors, except for the presence of a microcytic anemia probably due to HP-induced chronic atrophic gastritis.

The patient underwent a gastroscopy that confirmed a chronic gastritis with glandular atrophy, but that also revealed angiodysplasia of all the gastric mucosa and fundic gastric polyps.

Besides the known anemia, the initial blood tests showed also reduced protein levels, in particular albumin, probably due to the protein-losing gastropathy.

With regard to systemic risk factors, only the free protein S deficiency was found; this condition can be both congenital or acquired. In this particular case, it is unlikely that this deficiency was determined only by a congenital mechanism, considered the patient’s age and the absence of previous major thrombotic episodes. The deficit may be rather the consequence of a combination of two conditions, namely a mild congenital protein S deficiency worsened by the protein-losing gastropathy, as also described in Greenblatt and Nguyen’s case report [10].

With regard to PMVS thrombosis, the utility of thrombophilia testing is still a matter of debate [11]. Screening is indicated for patients with an episode of deep venous thromboembolism in an unusual site and in the absence of known local risk factors [12]. In the presented case, genetic tests were deemed unnecessary, considering the patient’s age and the absence of previous major thrombotic episodes. Nowadays, there are still no studies demonstrating the utility of thrombophilia lab testing to assess thromboembolism risk in individual patients [13] and some authors advocate to abandon routine laboratory screening [14].

Anticoagulant therapy should be continued for at least six months in patients without thrombogenic risk factors and be lifelong in those with persistent risk factors [15].

Quality improvement in imaging, a better understanding of the disease and early detection of acute SPMV thrombosis have reduced the need for surgery and, consequently, its mortality rate [16].

Indications for the surgical management of SPMV thrombosis are clinical findings of bowel infarction, perforation and peritonitis.

Intestinal infarction leads to bowel wall and mesenteric edema, as well as ascites production; reperfusion of the bowel can worsen parietal edema and ascites, increasing the risk for abdominal compartment syndrome.

For this reason, OA use should be considered after bowel reperfusion in AMI [17], in particular to facilitate second-look laparotomy to assess intestinal viability and, eventually, to perform bowel anastomosis [18]. In a setting of venous thrombosis, bowel resection is much less common than in a case of arterial occlusion, hence patients with mesenteric venous thrombosis probably do not require OA as often as those with acute arterial occlusion [19].

In the case reported here, a bowel resection was needed because of massive intestinal infarction.

Moreover, due to the certain in diagnosis at CT scan, a laparoscopic exploration was avoided in order to promptly perform resection and offer the patient a damage control resuscitation strategy in the ICU. The surgical strategy involved a second-look operation 48 h after the open abdomen management, as already described by Occhionorelli [20] and Burch [21]. A planned second-look operation remains the gold standard to assess bowel viability.

## 4 Conclusions

PMVS thrombosis is a rare condition that can lead to severe consequences like bowel infarction. It is usually determined by the combination of inherited and acquired risks factors, that should be carefully investigated to understand its pathogenesis and establish the adequate therapy.

The presence of intestinal infarction represents a mandatory indication for surgery. A planned surgical second-look, 24−48 hours after the open-abdomen management can be useful in assessing viability of the remnant bowel and feasibility of the anastomosis.

Patients should undergo lifelong anticoagulant therapy whenever in presence of persistent risk factors.

## Conflict of interest

None

## Funding

None.

## Ethical approval

Retrospective case reports do not require ethical approval.

## Consent

Written informed consent was obtained from the patient for publication of this case report and accompanying images.

Not essential identifying details were omitted.

## Author contribution

Annamaria Di Bella, MD: study concept and design, data collection, data analysis and interpretation, writing the paper, approval of the final version of the manuscript.

Alessandro Bruscino, MD: first operator in the surgical treatment described, study concept, data collection, approval of the final version of the manuscript.

Giovanni Alemanno, MD: study design, data analysis, approval of the final version of the manuscript.

Carlo Bergamini, MD: study concept, data analysis and interpretation, approval of the final version of the manuscript.

Paolo Prosperi, MD: data interpretation, approval of the final version of the manuscript.

## Registration of research studies

Retrospective not “first-in-men” case reports do not require registration.

NA

## Guarantor

Alessandro Bruscino, MD.

Paolo Prosperi, MD.

## Provenance and peer review

Not commissioned, externally peer-reviewed
